# Study of the therapeutic strategy to improve survival outcomes from the perspective of perioperative conditions in elderly gastric cancer patients: a propensity score-matched analysis

**DOI:** 10.1186/s12957-024-03488-1

**Published:** 2024-07-26

**Authors:** Eiji Nomura, Takatoshi Seki, Kentaro Yatabe, Hisamichi Yoshii, Hideki Izumi, Kazutake Okada, Hajime Kayano, Soichiro Yamamoto, Masaya Mukai, Hiroyasu Makuuchi

**Affiliations:** Department of Gastroenterological Surgery, Tokai University Hachioji Hospital, Hachioji, Tokyo 192-0032 Japan

**Keywords:** Gastric cancer, Elderly patient, Gastrectomy, Prognosis

## Abstract

**Background:**

Elderly gastric cancer patients (EGCPs) require treatment according to not just the stage of their cancer, but also to their general condition and organ function, and rather than full treatment, the appropriate amount of treatment is necessary.

**Methods:**

A total of 425 patients who underwent gastrectomy for primary gastric cancer in our institution between April 2013 and March 2020 were classified by age into two groups: elderly patients (EP, age ≥ 80 years, *n* = 89); and younger patients (YP, age < 80 years, *n* = 336). The preoperative, intraoperative, and postoperative conditions of the two groups were then compared. Propensity score matching (PSM) was performed, and factors affecting complications and survival outcomes were examined in detail. In addition, the necessary treatment strategy for EGCPs in the preoperative, intraoperative, and postoperative periods was investigated.

**Results:**

Of the preoperative factors, American Society of Anesthesiologists physical status (ASA-PS) was significantly higher, and respiratory function was significantly lower in the EP group than in the YP group, and the prognostic nutritional index (PNI) also tended to be lower. Of the intraoperative factors, there was no difference in the level of lymph node dissection. However, the EP group had significantly higher rates of postoperative pneumonia and anastomotic leakage. Of the postoperative factors, on simple comparison, postoperative long-term outcomes of the EP group were significantly worse (63.8% vs. 85.4%, *p* < 0.001), but there was no significant difference in disease-specific survival (DSS), and the DSS survival curves after PSM were almost identical, indicating that the survival rate in the EP group was decreased by death from other disease. Though the survival rate of laparoscopic surgery was significantly better than that of open surgery in the YP group, there was a significantly lower rate of postoperative complications in the EP group after PSM.

**Conclusions:**

In EGCPs, one needs to be aware of short-term complications such as pneumonia and anastomotic leakage due to respiratory dysfunction and malnutrition that are present before surgery. Furthermore, to suppress deaths from other diseases that reduce postoperative survival rates, prevention of postoperative complications (particularly pneumonia) through minimally invasive surgery can be effective.

## Background

In Japan, the reduction in the *Helicobacter pylori* infection rate is resulting in fewer gastric cancer patients [1]. Nevertheless, the dramatic aging of the country’s population means that the number of older people with gastric cancer is increasing, and, as a result, the absolute number of cases of gastric cancer is remaining steady [2]. This population aging also means that the number of gastric cancer patients with preoperative comorbidities is rising, so that a good deal of consideration must be given to surgery and perioperative management.

In Japan, treatment is provided in accordance with gastric cancer treatment guidelines [3], with D2 or greater lymph node dissection for advanced gastric cancer and D1 + lymph node dissection in principle for early gastric cancer. In terms of chemotherapy, postoperative adjuvant chemotherapy is recommended for histological Stage II/III patients, and chemotherapy regimens for Stage IV cases are also recommended. However, there is a tendency to limit lymph node dissection in patients who are older or at high risk [4], and whether chemotherapy is performed is also left to the discretion of the attending physician.

Patients aged ≥ 80 years were previously thought to be unsuitable for surgery and chemotherapy, but the rapidly increasing number of older gastric cancer patients and advances in medical science mean that treatment is now being actively provided even for these older patients [5]. Nevertheless, because aging is necessarily associated with reduced systemic organ function, treatment must take into account individual general condition and organ function, and rather than full treatment, it is the appropriate amount of treatment necessary that needs to be given. Nunobe et al. reported that the later perioperative mortality rate increased with increasing age, but they could not examine the effects of patient background in elderly Stage I patients, including comorbidities, on short- and long-term postoperative outcomes in a nationwide survey [6]. Matsunaga et al. reported that the disease-specific survival rate has decreased because sufficient D2 lymph node dissection and adjuvant chemotherapy cannot be performed in elderly patients with stage III gastric cancer [7]. They also emphasized the importance of nutritional support. On the other hand, Endo et al. stated the importance of performing surgery tailored to the patient’s condition by using indicators such as American Society of Anesthesiologists physical status (ASA-PS) as a reference [8]. Namely, even in elderly patients, the degree of cancer progression affects the prognosis; therefore, there are two schools of thought: one recommends radical surgery if possible, and the other recommends reduction surgery, emphasizing the safety of the surgery.

In this study, primary gastric cancer patients were classified as either elderly patients (EP) aged ≥ 80 years or younger patients (YP) aged < 80 years, and the preoperative, intraoperative, and postoperative conditions of each group were investigated. Propensity score matching (PSM) was also performed to reduce the effects of confounding factors, and then factors related to complications and survival outcomes were examined in detail. The purpose of this study was to clarify the preferred treatment strategy for elderly gastric cancer patients in the preoperative, intraoperative, and postoperative periods.

## Methods

This study involved 425 patients who underwent gastrectomy for primary gastric cancer in our institution during the seven-year period between April 2013 and March 2020. Patients with distant metastases that were preoperatively determined to be unresectable were excluded, as were those who underwent palliative surgery. The study patients were divided into the EP group of 89 patients and the YP group of 336 patients, and their preoperative patient factors [age, sex, ASA-PS, respiratory function (%VC, %FEV1.0), and inflammation-based factors], intraoperative patient factors (pathological gastric wall invasion depth (pT), pathological level of lymph node metastasis (pN), histological type, pathological stage (pStage), type of gastrectomy, lymph node dissection level, intraoperative hemorrhage, and operating time), and postoperative patient factors [postoperative chemotherapy, disease-specific survival (DSS), death from other disease, survival rate] were compared between the two groups. The survival rate was calculated as the three-year survival rate until March 2023. Blood samples were collected within 1 month prior to surgery, and the neutrophil-to-lymphocyte ratio (NLR) and the platelet-to-lymphocyte ratio (PLR) were calculated as inflammation-based factors [9]. The C-reactive protein-to-albumin ratio (CAR) [10] and the prognostic nutritional index (PNI) [11] were also calculated. To minimize selection bias between the two groups, PSM was performed with a logistic regression model and 1:1 nearest neighbor-matching using JMP for Windows version 13.0 (SAS Institute, Cary, NC) software. The following variables were selected as matching variables because these variables were determined to have a significant survival impact: pathological depth of tumor invasion (T1, T2, T3, T4), pathological lymph node metastasis (N0, N1, N2, N3), histological type (differentiated, undifferentiated), pathological stage (pStage I, II, III, IV), type of gastrectomy (distal gastrectomy, total gastrectomy, proximal gastrectomy), and lymphadenectomy (D0-1, D1+, D2, D2+-3). Next, factors affecting survival outcomes in all cases were analyzed using univariate and multivariate analyses, and analyses were also conducted in the EP group, YP group, and PSM group. In this way, findings characteristic of the EP group compared with the YP group were extracted, the factors that affected survival outcomes were examined, and treatment strategies to improve the prognosis of the EP group were evaluated.

This study protocol was approved by the Human Ethics Review Committee of Tokai University School of Medicine (Institutional Review Board Number 23R047).

Clinicopathological findings of the gastric resections were recorded according to the Japanese Classification of Gastric Carcinoma, 3rd English edition [12].

Differences between the two groups were tested for significance using the χ^2^ test, *t*-test, and Mann-Whitney’s U test. Overall survival in the two groups was calculated by the Kaplan-Meier method, and the difference was tested using the log-rank test. To identify prognostic factors, univariate analysis was performed first, followed by multivariate analysis using all significant variables from univariate analysis. Multivariate analysis was performed using logistic regression. The software used for all statistical analyses was JMP for Windows version 13.0 (SAS Institute, Cary, NC). Cut-off values are shown in Table 5. In all cases, *p* < 0.05 was regarded as significant.

## Results

### Patients’ background characteristics (preoperative factors)

Table 1 shows a comparison of preoperative patient factors between the EP and YP groups. Men outnumbered women in both groups, but there was a higher proportion of women in the EP group than in the YP group. Both ASA-PS and respiratory function (%VC, %FEV1.0) were significantly worse in the EP group than in the YP group, the NLR was higher, and the PNI was lower. Even after PSM, ASA-PS and respiratory function were lower in the EP group, and the PNI also tended to be lower.

**Table 1:** Patients’ background characteristics and preoperative data

Characteristic	Before matching			After matching		
	EP group (*n* = 89)	YP group (*n* = 336)	*P* value	EP group (*n* = 80)	YP group (*n* = 80)	*P* value
Age (y)	83.2 ± 2.6	67.6 ± 8.7	< 0.001	83.1 ± 2.7	67.7 ± 9.1	< 0.001
Sex			0.025			0.526
Male	52(58.4)	238(70.8)		45 (56.3)	41 (51.3)	
Female	37(41.6)	98(29.2)		35 (43.7)	39 (48.7)	
ASA-PS			< 0.001			0.008
1	2(2.2)	22((6.5)		2 (2.5)	5 (6.3)	
2	53(59.6)	262(78.0)		51 (63.8)	64 (80.0)	
3	32(36.0)	50(14.9)		26 (32.5)	11 (13.7)	
4	2(2.2)	2(0.6)		1 (1.2)	0 (0)	
%VC	91.9 ± 19.0	103.6 ± 17.1	< 0.001	92.4 ± 19.4	105.4 ± 15.6	< 0.001
%FEV1.0	71.2 ± 10.7	75.8 ± 9.1	< 0.001	71.4 ± 10.8	75.7 ± 9.3	0.008
NLR	2.80 ± 1.60	2.34 ± 1.54	0.014	2.73 ± 1.56	2.47 ± 2.11	0.373
PLR	156.2 ± 73.4	141.8 ± 67.5	0.080	158.8 ± 71.1	146.0 ± 82.7	0.297
CAR	0.20 ± 0.49	0.16 ± 0.74	0.588	0.21 ± 0.51	0.13 ± 0.27	0.243
PNI	44.1 ± 7.3	49.1 ± 22.1	0.036	44.3 ± 7.3	52.6 ± 43.7	0.098

ASA-PS: American Society of Anesthesiologists-physical status, %VC: (measured vital capacity/predictive vital capacity) × 100 (%), %FEV1.0: (forced expiratory volume in one second/forced vital capacity) × 100 (%), NLR: neutrophil-to-lymphocyte ratio, PLR: platelet-to-lymphocyte ratio, CAR: C-reactive protein-to-albumin ratio, PNI: prognostic nutritional index (10× serum albumin (g/dL) + 0.005× lymphocytes (/µL))

### Short-term results (intraoperative factors)

Table 2 shows a comparison of intraoperative patient factors. Wall invasion depth and level of lymph node metastasis were both higher in the EP group than in the YP group, and pStage was also more severe. In terms of the type of gastrectomy, open surgery was more common than laparoscopic surgery in the EP group, and distal gastrectomy was the most common procedure, with few other types of gastrectomy conducted. Limited surgery, which was defined as less than D2 lymphadenectomy by the gastric cancer treatment guideline [3], was performed for 70.8% of the EP group and 56.9% of the YP group. Although D1 + lymph node dissection was somewhat more common than D2 lymph node dissection in the EP group, there was no overall difference in the lymph node dissection level. In addition, three high-risk cases who developed bleeding (one in the EP group and two in the YP group) underwent gastrectomy (D0-D1) as for a gastric ulcer.

**Table 2:** Pathological findings, operative procedures, and intraoperative findings

Characteristic	Before matching			After matching		
	EP group (*n* = 89)	YP group (*n* = 336)	*P* value	EP group (*n* = 80)	YP group (*n* = 80)	*P* value
Depth of invasion			0.047			0.838
T1	34 (38.2)	181 (53.9)		34 (42.5)	35 (43.8)	
T2	18 (20.2)	41 (12.2)		15 (18.8)	17 (21.3)	
T3	21 (23.6)	63 (18.8)		19 (23.8)	15 (18.8)	
T4	16 (18.0)	51 (15.1)		12 (14.9))	13 (16.1)	
Lymph node metastasis			0.032			0.959
N0	46 (51.7)	223 (66.4)		44 (55.0)	43 (53.8)	
N1	12 (13.5)	39 (11.6)		11 (13.8)	12 (15.0)	
N2	17 (19.1)	31 (9.2)		14 (17.4)	15 (18.8)	
N3	14 (15.7)	43 (12.8)		11 (13.8)	10 (12.4)	
Histological type			0.385			0.521
Differentiated	52 (58.4)	179 (53.3)		49 (61.3)	45 (56.3)	
Undifferentiated	37 (41.6)	157 (46.7)		31 (38.7)	35 (43.7)	
pStage			0.038			0.537
I	42 (47.2)	201 (59.8)		40 (50.0)	41 (51.3)	
II	20 (22.5)	64 (19.0)		19 (23.8)	16 (20.0	
III	20 (22.5)	60 (17.9)		17 (21.2)	17 (21.3)	
IV	7 (7.8)	11 (3.3)		4 (5.0)	6 (7.4)	
Approach			0.007			0.752
Open	47 (52.8)	124 (36.9)		39 (48.8)	41 (51.2)	
Laparoscopic	42 (47.2)	212 (63.1)		41 (51.2)	38 (48.8)	
Type of gastrectomy			0.013			0.751
Distal	71 (79.8)	212 (63.1)		63 (78.8)	65 (81.3)	
Total	14 (15.7)	89 (26.5)		13 (16.2)	13 (16.2)	
Proximal	4 (4.5)	35 (10.4)		4 (5.0)	2 (2.5)	
Lymphadenectomy			0.981			0.893
D0-1	1 (1.1)	2 (0.6)		1 (1.3)	1 (1.3)	
D1+	62 (69.7)	189 (56.3)		54 (67.5)	53 (66.3)	
D2	26 (29.2)	144 (42.8)		25 (31.2)	26 (32.4)	
D2+-3	0 (0.0)	1 (0.3)		0 (0.0)	0 (0.0)	
Blood loss (mL)	250.1 ± 295.5	285.0 ± 452.4	0.491	245.4 ± 296.7	218.5 ± 293.8	0.565
Operation time (h)	266.2 ± 64.3	315.2 ± 60.8	< 0.001	270.1 ± 62.1	296.4 ± 51.3	0.004

pStage: pathological stage, Distal: distal gastrectomy, Total: total gastrectomy, Proximal: proximal gastrectomy, Open: open gastrectomy, Laparoscopic: laparoscopic gastrectomy

Furthermore, operating time was significantly shorter in the EP group, before and after PSM.

### Long-term results (postoperative factors)

In the comparison of postoperative patient factors (Table 3), a significantly smaller proportion of patients received chemotherapy in the EP group than in the YP group, and this was still the case after PSM. In addition, the rate of adjuvant chemotherapy for pStage II and III was significantly lower in the EP group than in the YP group (20.0% (8/40) vs. 71.8% (89/124), *P* < 0.001). In the EP group, there was a significant tendency for a higher number of deaths from other conditions, and this was still the case after PSM. Regarding early postoperative complications (Table 4), the rates of postoperative pneumonia and other complications were both higher in the EP group than in the YP group. After PSM, however, in addition to postoperative pneumonia, a significant difference in the incidence of anastomotic leakage was also apparent.

**Table 3:** Postoperative therapy and death from primary and other diseases (postoperative data)

Characteristic	Before matching			After matching		
	EP group (*n* = 89)	YP group (*n* = 336)	*P* value	EP group (*n* = 80)	YP group (*n* = 80)	*P* value
Adjuvant chemotherapy			< 0.001			< 0.001
Present	11 (11.4)	107 (31.8)		9 (11.3)	37 (46.3)	
Absent	78 (87.6)	229 (68.2)		71 (88.7)	43 (53.7)	
Death from primary disease			0.519			0.405
Present	16 (18.0)	51 (15.2)		12 (15.0)	16 (20.0)	
Absent	73 (82.0)	285 (84.8)		68 (85.0)	64 (80.0)	
Death from other diseases			< 0.001			0.015
Present	14 (15.7)	15 (4.5)		12 (15.0)	1 (1.3)	
Absent	75 (84.3)	321 (95.5)		68 (85.0)	79 (98.7)	

Primary disease: gastric cancer

**Table 4:** Early postoperative complications

Characteristic	Before matching			After matching		
	EP group (*n* = 89)	YP group (*n* = 336)	*P* value	EP group (*n* = 80)	YP group (*n* = 80)	*P* value
Pancreatic fistula	5 (5.6)	18 (5.4)	0.923	4 (5.0)	5 (6.3)	0.732
Anastomotic leakage	4 (4.5)	7 (2.1)	0.203	4 (5.0)	0 (0)	0.043
Anastomotic stenosis	1 (1.1)	4 (1.2)	0.959	1 (1.3)	1 (1.3)	1
Anastomotic ulcer	1 (1.1)	1 (0.3)	0.311	1 (1.3)	0 (0)	0.316
Lymphorrhea	1 (1.1)	1 (0.3)	0.311	1 (1.3)	0 (0)	0.316
Hemorrhage, abscess	2 (2.2)	6 (1.8)	0.776	2 (2.5)	1 (1.3)	0.560
Cholecystitis	1 (1.1)	2 (0.6)	0.597	1 (1.3)	0 (0)	0.316
Pneumonia	4 (4.5)	1 (0.3)	0.001	4 (5.0)	0 (0)	0.043
Others	9 (10.1)	5 (1.5)	< 0.001	3 (3.8)	0 (0)	0.080
Heart failure	2 (2.2)	3 (0.9)	0.292	1 (1.3)	0 (0)	0.316
Cerebral infarction	3 (3.4)	0 (0)	< 0.001	1 (1.3)	0 (0)	0.316
Renal infarction	1 (1.1)	1 (0)	0.311	0 (0)	0 (0)	―
Hepatorenal failure	1 (1.1)	1 (0)	0.311	0 (0)	0 (0)	―
Convulsion	1 (1.1)	0 (0)	0.052	0 (0)	0 (0)	―
LL arterial occlusion	1 (1.1)	0 (0)	0.052	1 (1.3)	0 (0)	0.316
Total	28 (31.5)	45 (13.4)	< 0.001	21 (26.3)	7 (8.8)	0.004

LL: lower leg

Next, univariate and multivariate analyses were performed to examine factors related to survival using all preoperative, intraoperative, and postoperative factors, and the results of the examination for all cases are shown in Table 5. In addition, pT and pN were excluded from the study items because they are included in pStage. Only pStage, PNI, and surgical approach were found to be significant independent factors, and a trend was observed in ASA-PS. However, on multivariate analysis of the YP group alone, only pStage remained, and no significant survival-related factors were found in the EP group. Among all cases in which PSM was performed, only pStage was extracted as a significant factor related to survival.

**Table 5:** Univariate and multivariate analyses of prognostic factors in all patients

Characteristic	Univariate analysis			Multivariate analysis		
	Hazard ratio	95%CI	*P* value	Hazard ratio	95%CI	*P* value
Age( ≧ 80 vs. < 80)	2.120	1.265–3.55	0.062			
Sex(Male vs. Female)	1.148	0.698–1.889	0.619			
ASA-PS(1,2 vs. 3,4)	0.293	0.175–0.488	< 0.001	0.565	0.288–1.109	0.097
%VC(> 80 vs. ≤ 80)	0.327	0.176–0.608	< 0.001	0.581	0.256–1.319	0.194
%FEV1.0(> 70 vs. ≤ 70)	0.728	0.438–1.212	0.227			
NLR(< 2.44 vs. ≥ 2.44)	0.786	0.494–1.251	0.337			
PLR(< 145 vs. ≥ 145)	0.750	0.474–1.187	0.239			
CAR(< 0.165 vs. ≥ 0.165)	0.299	0.173–0.515	< 0.001	0.905	0.441–1.856	0.786
PNI((< 48 vs. ≥ 48)	0.308	0.188–0.504	< 0.001	0.471	0.257–0.862	0.015
Histological type (Diff. vs. Undiff.)	0.736	0.466–1.162	0.200			
pStage(I,II vs III, IV)	0.081	0.048–0.139	< 0.001	0.156	0.076–0.321	< 0.001
Approach(Open vs. laparoscopic)	7.304	4.32–12.350	< 0.001	2.308	1.018–5.234	0.045
Type of gastrectomy (Distal, Proximal vs. Total)	0.418	0.255–0.684	< 0.001	1.073	0.563–2.045	0.830
Lymphadenectomy(D0-1, D1 + vs. D2, D2+-3)	0.493	0.311–0.782	0.003	0.830	0.624–2.347	0.573
Blood loss(< 280 vs. ≥ 280)	0.231	0.143–0.373	< 0.001	0.982	0.465–2.076	0.962
Op. time(< 300 vs. ≥ 300)	1.316	0.833–2.081	0.247			
Adj. chemo. (absent vs. present)	0.297	0.184–0.480	< 0.001	0.843	0.411–1.723	0.640
Postop. Complication (absent vs. present)	0.458	0.256–0.818	0.013	0.620	0.297–1.292	0.202

ASA-PS: American Society of Anesthesiologists-physical status, %VC: (measured vital capacity/predictive vital capacity) × 100 (%), %FEV1.0: (forced expiratory volume in one second/forced vital capacity) × 100 (%), NLR: neutrophil-to-lymphocyte ratio, PLR: platelet-to-lymphocyte ratio, CAR: C-reactive protein-to-albumin ratio, PNI: prognostic nutritional index (10× serum albumin (g/dL) + 0.005× lymphocytes (/µL)), Diff: differentiated, Undiff: Ubdifferentiated, pStage: pathological stage, Distal: distal gastrectomy, Total: total gastrectomy, Proximal: proximal gastrectomy, Open: open gastrectomy, Laparoscopic: laparoscopic gastrectomy, Op.: operation, Adj. chemo.: Adjuvant chemotherapy, Postop.: postoperative

**Fig. 1:**
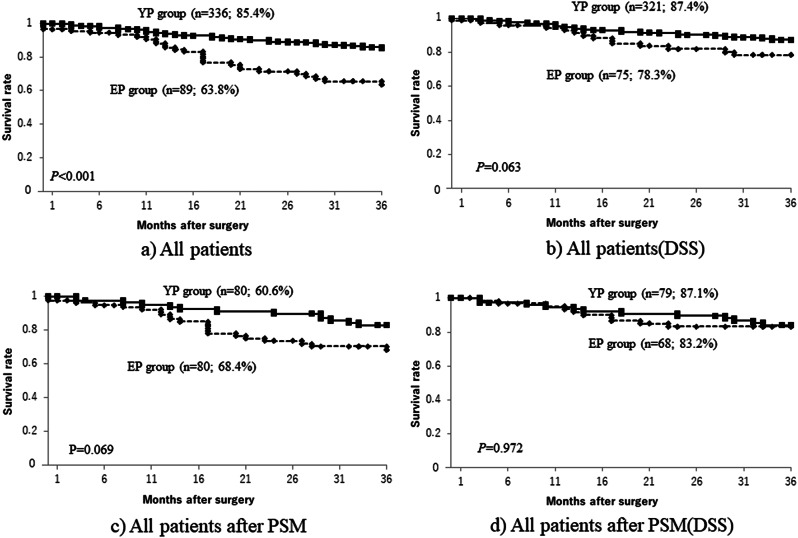
Overall survival curves for all patients (**a**) and those after PSM (**c**), and disease-specific survival curves of all patients (**b**) and those after PSM (**d**) PSM: propensity score matching

**Fig. 2:**
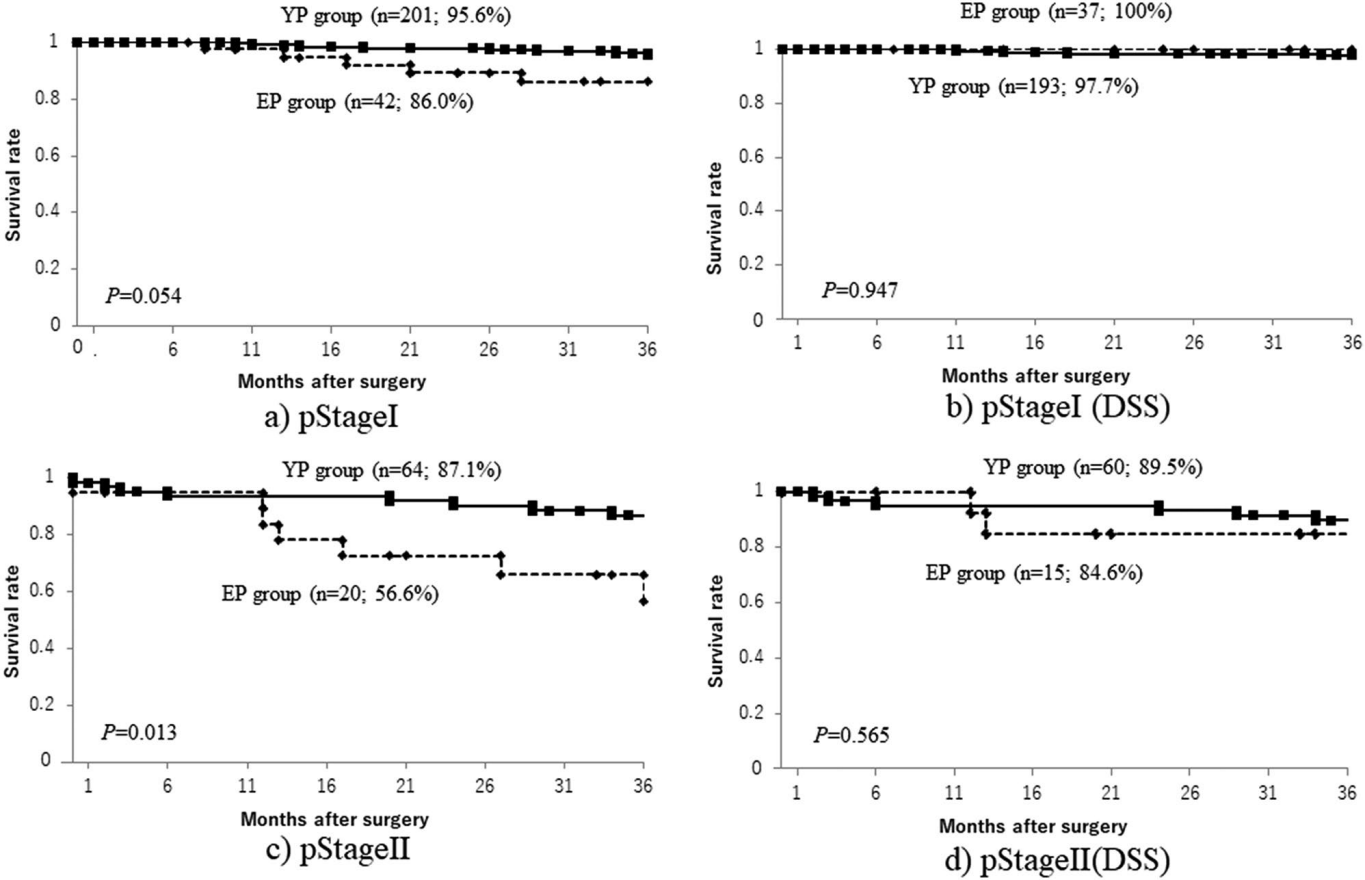
Overall survival curves for pStage I (**a**) and pStage II (**c**), and disease-specific survival curves for pStage I (**b**) and pStage II (**d**)

**Fig. 3:**
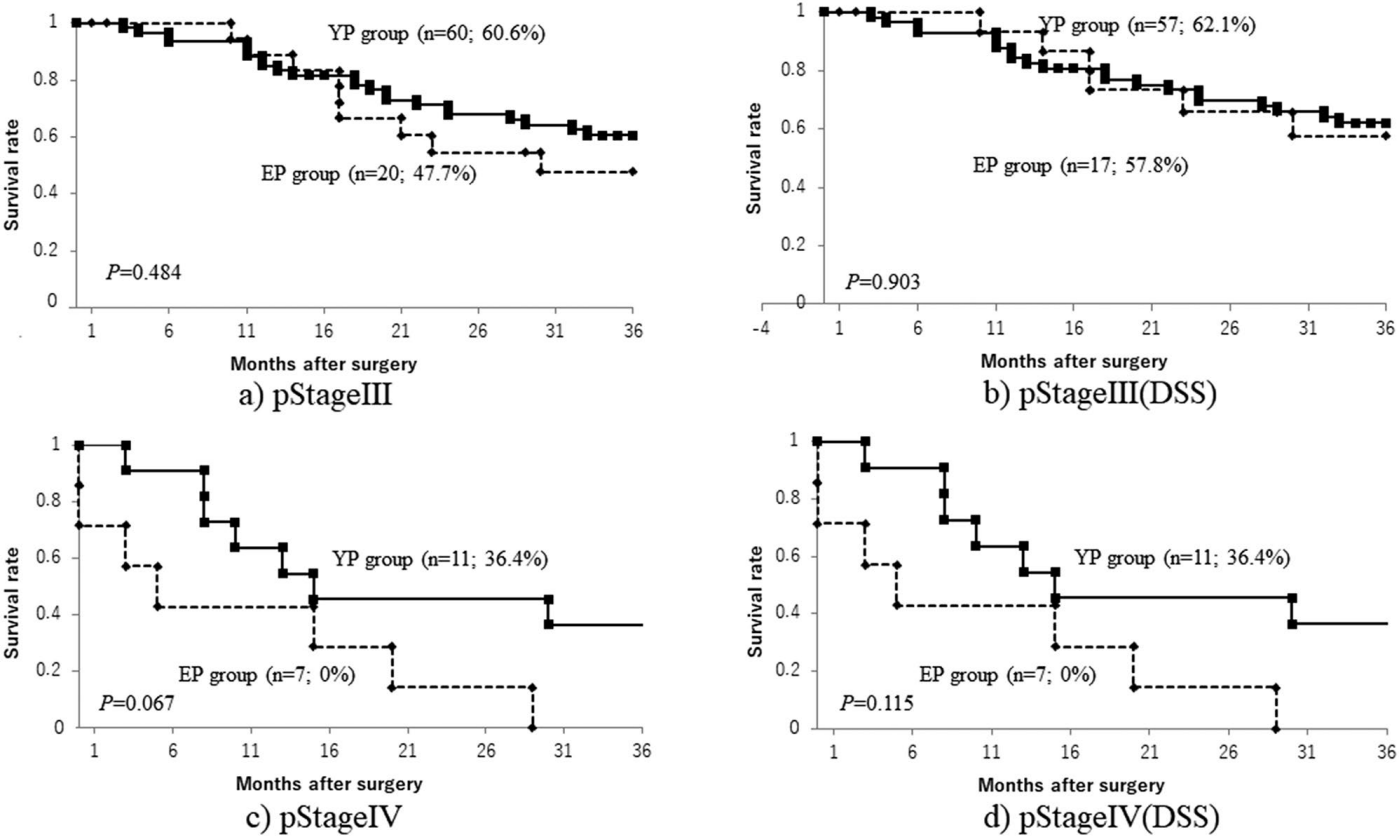
Overall survival curves for pStage III (**a**) and pStage IV (**c**), and disease-specific survival curves for pStage III (**b**) and pStage IV (**d**)

A comparison of cumulative survival rates in the EP and YP groups showed that, though long-term outcomes were significantly worse in the EP group than in the YP group (Fig. 1a), when death from other disease was excluded, DSS tended to be lower in the EP group, but the difference between the two groups was not significant (Fig. 1b). A comparison by pStage (Figs. 2 and 3) showed that the only significant difference was for pStage II patients (Fig. 2c), and that this, too, was no longer significant after deaths from other diseases were excluded (Fig. 2d). After PSM, cumulative survival rates also tended to be somewhat lower in the EP group than in the YP group (Fig. 1c), but after deaths from other diseases were excluded, the survival curves were almost identical (Fig. 1d). This suggested that death from other disease may have been the main factor decreasing the survival rate in the EP group compared with that in the YP group, especially in Stages I and II.

A comparison of cumulative survival rates in the EP and YP groups shows that, though long-term outcomes are significantly worse for the EP group than for the YP group, when deaths from other diseases are excluded, although disease-specific survival (DSS) tends to be lower in the EP group, the difference between the two groups is not significant. After PSM, cumulative survival rates also tend to be somewhat lower in the EP group than in the YP group, but once deaths from other diseases are excluded, the survival curves are almost identical

A comparison by pStage shows that the only significant difference is for pStage II patients, but this is no longer significant after deaths from other diseases are excluded

The underlying diseases present in those patients who died from the primary cancer and those who died from another disease were therefore investigated by identifying serious diseases that had previously required invasive treatment and classifying them as cardiovascular disease, cerebrovascular disease, respiratory disease, or other disease, as shown in Table 6. In both groups, more patients who died from another disease had a serious underlying disease than those who died from the primary cancer, and in particular, the rates of cerebrovascular and cardiovascular diseases were significantly higher in the YP group. The rates of cerebrovascular and cardiovascular diseases also tended to be higher in the EP group, but many other comorbidities were also present. However, when the final cause of death was examined in patients who died from other diseases, respiratory diseases were the most common cause of death in the EP group (Table 7).

**Table 6:** Comparison of underlying diseases between patients who died from primary disease and other diseases in the EP and YP groups

Severe underlying disease	EP group		*P* Value	YP group		*P* Value
	Death from primary disease (%)	Death from other diseases (%)		Death from primary disease (%)	Death from other diseases (%)	
Cardiovascular disease	4 (25.0)	5 (35.7)	0.070	3 (5.9)	5 (33.3)	0.025
Cerebrovascular disease	1 (6.3)	4 (28.6)		4 (7.8)	1 (6.7)	
Respiratory disease	0 (0)	0 (0)		1 (2.0)	3 20.0)	
Other	1 (6.3)	5 (35.7)		4 (7.8)	0 (0)	
Liver failure (Cirrhosis)	0 (0)	2 (14.3)	0.028	3 (5.9)	0 (0)	0.002
Renal failure (Dialysis)	1 (6.3)	1 (7.1)		2 (3.9)	0 (0)	
Pancreatic cancer	0 (0)	1 (7.1)		0 (0)	0 (0)	
Collagen disease	0 (0)	1 (7.1)		0 (0)	0 (0)	
None	11 (68.8)	4 (28.6)		41 (80.4)	6 (40.0)	
Total	16 (100)	14 (100)		51 (100)	15 (100)	

(Includes multiple diseases)

**Table 7:** Comparison of causes of death between the EP group and the YP group in patients who died from other diseases

Cause of death	EP group (%)	YP group (%)	*P* Value
Cardiovascular disease	2 (14.3)	3 (20.0)	0.684
Cerebrovascular disease	2 (14.3)	2 (13.3)	0.941
Respiratory disease	5 (35.7)	1 (6.7)	0.054
Others	5 (35.7)	9 (60.0)	0.191
Total	14 (100)	15 (100)	

**Fig. 4:**
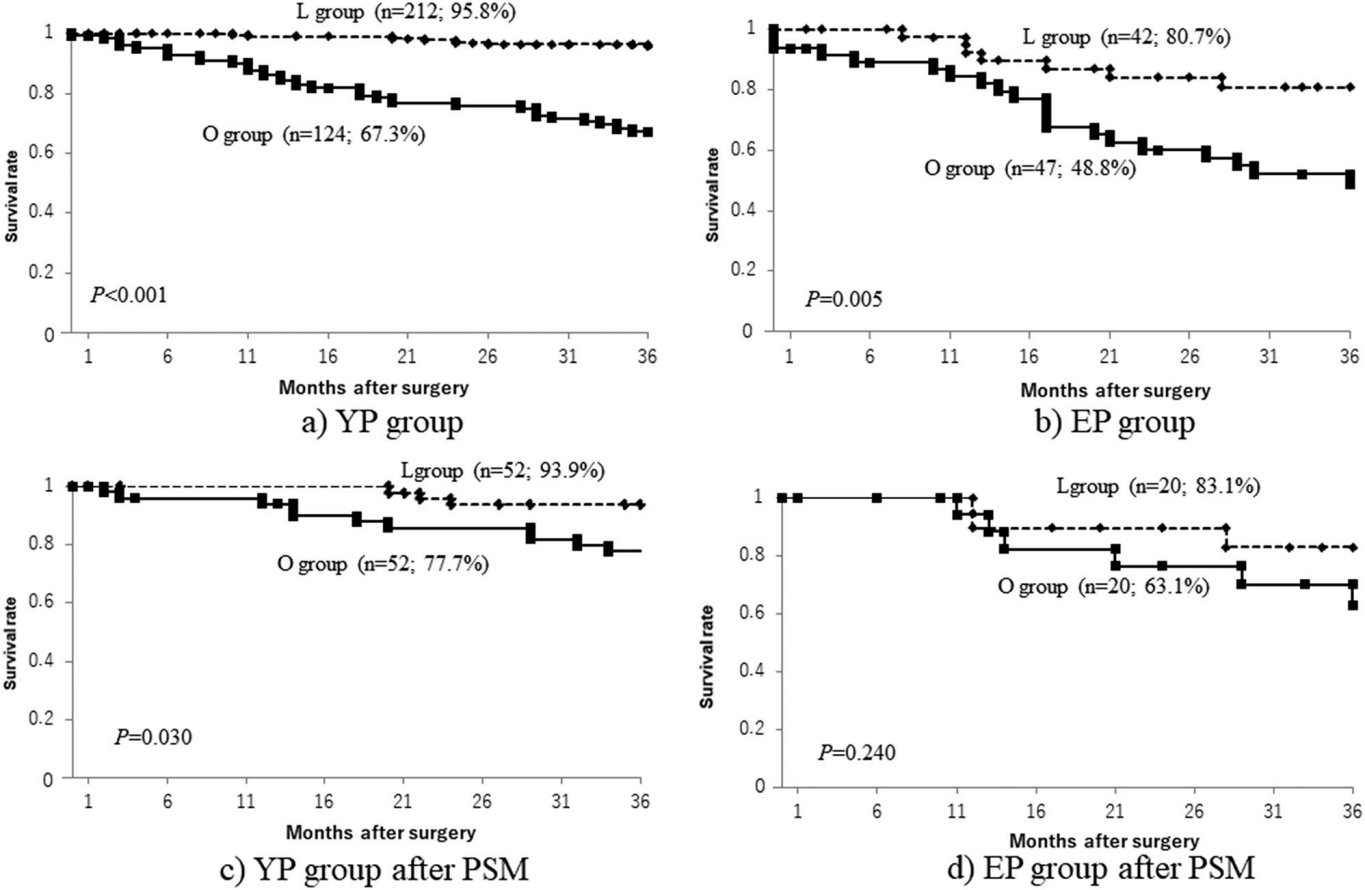
Overall survival curves for the YP group (**a**) and the EP group (**b**), the YP group after PSM (**c**) and the EP group after PSM (**d**) PSM: propensity score matching, L: laparoscopic gastrectomy, O: Open gastrectomy In both the EP and YP groups, the L group has a significantly higher survival rate than the O group. Even with PSM, in the YP group the survival rate of the L group is significantly better than that of the O group, but in the EP group, there is no significant difference in the survival rate between the L group and the O group

Finally, when comparing the survival rates between open surgery (O group) and laparoscopic surgery (L group), which had significantly different survival outcomes on multivariate analysis, it was found that the L group had a significantly higher survival rate than the O group in both the EP and YP groups (Fig. 4a, b). However, even after PSM, in the YP group, the survival rate of the L group was significantly better than that of the O group (Fig. 4c), and when the numbers of complications were investigated, there was a tendency for a lower incidence rate of postoperative complications (Table 8A). However, in the EP group after PSM, there was no significant difference in the survival rate between the L group and the O group (Fig. 4d), but the rate of postoperative complications was significantly lower (Table 8B).

**Table 8:** Comparison of postoperative complications between open and laparoscopic gastrectomies after PSM in the YP group (**A**) and the EP group (**B**)

YP group (A)	Open (*n* = 52)	Laparoscopic (*n* = 52)	*P* value
Pancreatic fistula	3 (5.8)	2 (3.8)	0.647
Anastomotic leakage	2 (3.8)	1 (1.9)	0.558
Hemorrhage	1 (1.9)	0 (0)	0.315
Pneumonia	1 (1.9)	0 (0)	0.315
Cholecystitis	1 (1.9)	0 (0)	0.315
Lymphorrhea	0 (0)	1 (1.9)	0.315
Others	2 (3.8)	0 (0)	0.153
Hepatorenal failure	1 (1.9)	0 (0)	0.315
Renal failure	1 (1.9)	0 (0)	0.315
Total	10 (9.2)	4 (7.7)	0.085
**EP group (B)**	Open (*n* = 20)	Laparoscopic (*n* = 20)	***P*** ** value**
Pancreatic fistula	2 (10.0)	0 (0)	0.147
Anastomotic leakage	1 (5.0)	0 (0)	0.311
Anastomotic stricture	1 (5.0)	0 (0)	0.311
Hemorrhage	1 (5.0)	0 (0)	0.311
Pneumonia	0 (0)	1 (0)	0.311
Cerebral infarction	1 (5.0)	0 (0)	0.311
Total	6 (30.0)	1 (5.0)	0.037

PSM: propensity score matching, Open: open gastrectomy, Laparoscopic: laparoscopic gastrectomy

## Discussion

In this study, primary gastric cancer patients were classified into the EP group, aged ≥ 80 years, and the YP group, aged < 80 years. The significantly higher proportion of women in the EP group was thought to be due to women’s longer average lifespan [13]. However, Arakawa et al. also stated that male gastric cancer patients aged 75 years or older have more upper gastric cancer, more frequent postoperative complications, and a worse prognosis than female patients [14]. Furthermore, the EP group was characterized by poor performance status, respiratory function, immune function, and nutritional condition. The investigation of operative factors identified a significantly higher rate of laparoscopic surgery in the YP group, but no such significant difference was evident in the EP group. This may have been both because the YP group included more Stage I cases, and because more patients in the EP group had preoperative comorbidities that made it desirable to reduce operating time. However, in the era when only open surgery was performed, shortening the operation time might have been an important factor in making the procedure less invasive, but this concept may no longer apply now that laparoscopy has become commonplace.

The present examination of the type of gastrectomy showed that there were fewer total gastrectomies in the EP group, and that, though there was no significant difference in the lymph node dissection level, fewer D2 lymph node dissections were performed. Kiuchi et al. reported that postoperative pneumonia was associated with poor long–term outcomes in patients with gastric cancer [15]. Suzuki et al. stated that overall survival was poor for patients aged ≥ 75 years with ASA-PS scores of 3 who underwent D2 lymph node dissection, and that postoperative pneumonia was implicated in their poor prognosis, with D2 lymph node dissection being a significant risk factor for postoperative pneumonia [16]. Kimura et al. also showed that D2 lymph node dissection was an independent risk factor for postoperative pneumonia in 75-year-old patients [17]. In the present study, the incidence of postoperative pneumonia was significantly higher in the EP group than in the YP group. Based on preoperative data, the incidence of pneumonia may have been higher because respiratory function in the EP group was already significantly lower preoperatively, and there was no difference in lymph node dissection level.

The tendency for PNI to be lower after PSM indicates that nutritional status in the EP group was poor, and this may have increased the incidence of anastomotic leakage. The present result that PNI was identified as one of the factors contributing to survival on multivariate analysis that included all factors also suggests that poor nutritional status can trigger these complications and affect survival rates. This seems to support the possibility that there is a relationship. Rosenberg et al. defined sarcopenia as age-related loss of muscle mass [18], but secondary sarcopenia may also occur as a result of causes such as reduced activity, disease, and malnutrition [19], and older gastric cancer patients may have both these forms of sarcopenia simultaneously. Fukuda et al. reported that preoperative sarcopenia was a risk factor for severe postoperative complications in gastric cancer patients aged ≥ 65 years [20], whereas Wang et al. stated that preoperative sarcopenia and diabetes mellitus are predictors of complications after gastric cancer surgery [21]. In the present study, the EP group also included a higher proportion of patients who had a high ASA-PS score. This means that, because the sarcopenia-like condition suffered by older gastric patients may cause postoperative complications, particularly anastomotic leakage, a range of perioperative nutritional therapies is being used [22, 23].

However, cancer stage also affects the prognosis of older patients, and though some studies recommend proactively conducting curative surgery [24, 25], others encourage the use of less invasive procedures that prioritize operative safety to prevent postoperative complications [8, 26]. Konishi et al. reported that DSS was significantly higher in older patients with cStage II gastric cancer who underwent curative gastrectomy with lymph node dissection [27]. Matsunaga et al. also stated that older Stage III gastric cancer patients (≥ 75 years old) had significantly poorer DSS than younger gastric cancer patients (≤ 74 years), and that this was due to the former’s poor nutritional status and immune function, as well as their lower rates of D2 lymph node dissection and adjuvant chemotherapy [7]. However, the present data did not show a difference in DSS between patients at different stages, suggesting that it may be better to minimize surgical invasion as much as possible. Arakawa et al. reported that reduction surgery without postoperative complications had a better prognosis than standard surgery with postoperative complications [14]. The JLSSG0901 study demonstrated that laparoscopic distal gastrectomy with D2 lymph node dissection is not inferior to open distal gastrectomy for locally advanced gastric cancer [28]. Laparoscopic surgery also reportedly reduces the incidence of a range of complications than open surgery for gastric cancer patients in poor general condition with ASA-PS ≥ 3 [29]. Tanaka et al. used PSM to show that laparoscopic surgery shortened the length of hospital stay and reduced postoperative complications in gastric cancer patients aged ≥ 80 years compared with open surgery [30]. In the present study, laparoscopic surgery also improved the survival rate in the YP group and tended to reduce postoperative complications. Although there was no improvement in the survival rate after PSM in the EP group, there was a significant decrease in postoperative complications.

However, the death rate was significantly higher in the EP group, and this was characterized by the occurrence of more deaths from other diseases. The significant difference in the survival rate seen in stage II patients in the EP group also disappeared after death from other diseases was excluded. Kakeji et al. not only showed that both 5-year overall survival (OS) and 5-year DSS were poor in patients aged ≥ 80 years, but they also showed that there was a large difference in both OS and DSS between patients aged ≥ 80 years and those aged < 80 years, and that death from other diseases had a major effect above the age of 80 years [31].

However, although underlying disease was investigated with the aim of identifying factors causing death from other diseases in older patients, numerous significant, serious, underlying diseases were present in those who died from other diseases, with cerebrovascular and cardiovascular diseases being particularly common. This tendency was more pronounced in the YP group. However, the final cause of death from other diseases did not necessarily correspond to these underlying diseases, and it tended to correspond to respiratory disease more frequently in the EP group. From this, it is thought that, in elderly patients, attention should be paid to respiratory complications associated with decreased respiratory function in the early postoperative period and also in the long term postoperatively. Kamiya et al. reported that death from other diseases is not related to preoperative complications, but rather to postoperative complications and open gastrectomy [32]. Laparoscopic surgery, which reduces postoperative complications, is likely to be beneficial in reducing the number of deaths from other diseases, which has decreased the survival rate of elderly patients, as shown in the results. Although the benefit of laparoscopy was not demonstrated in the EP group, it is thought that, by proactively introducing minimally invasive surgery in the future, the survival rate will improve, as in the YP group.

Despite the significantly higher number of patients at a high pStage in the EP group, fewer of these patients underwent postoperative adjuvant chemotherapy, which may have been because of their lower postoperative performance status due to age-related underlying conditions and reduced organ function [33]. The merits and disadvantages of adjuvant chemotherapy for older gastric cancer patients are the subject of debate. Wakahara et al. recommended active treatment, such as surgery and adjuvant chemotherapy, if possible, and reported improved survival in older adult patients with advanced gastric cancer who received adjuvant chemotherapy for > 3 months [34]. Meanwhile, Schendel et al. reported that surgery alone improved survival compared to conservative treatment in older adult patients who were ineligible to receive chemotherapy [35]. The present data also showed that, since only 20.0% of patients in the EP group underwent adjuvant chemotherapy, its efficacy was difficult to evaluate, but it may be better to consider the use of adjuvant chemotherapy in patients not at risk of death from other disease, that is, those with good ASA-PS and no pre-existing comorbidities such as cerebrovascular or cardiovascular diseases, rather than on the basis of age. There is little evidence for the use of adjuvant chemotherapy in older gastric cancer patients, but in the ACTS-GC clinical trial [36], which demonstrated the efficacy of S-1 adjuvant chemotherapy for Stage II or III gastric cancer, patients aged ≥ 80 years were excluded. Phase III clinical trials to confirm the value of modified S-1 adjuvant chemotherapy following gastrectomy for frail pStage II/III older gastric cancer patients (JCOG1507, BIRDIE) are currently underway [37], and their results are awaited.

This study had a number of inherent limitations. The first was its retrospective nature. Second, follow-up was insufficient for some patients. Third, it was conducted as a single-center study of a comparatively small number of patients. Further prospective studies involving more patients in more institutions are desirable in the future. In particular, for ASA-PS, which showed a tendency to be associated with mortality on multivariate analysis of all cases, prospective studies involving a larger number of elderly patients are needed.

In conclusion, older gastric cancer patients aged ≥ 80 years may have sarcopenia associated with poor nutrition and decreased immune function. This means every effort should be made to improve their preoperative nutritional status as much as possible to prevent anastomotic leakage, and since their respiratory function is also decreased, it is also necessary to attempt minimally invasive surgery with D1 + lymph node dissection so that surgery is neither excessive nor insufficient. Beyond these efforts, it is believed that it will be possible to prevent death from other diseases that reduce the survival rate of elderly gastric cancer patients.

## Data Availability

No datasets were generated or analysed during the current study.

## Abbreviations

EP: Elderly patient
YP: Younger patient
PSM: Propensity score matching
ASA-PS: American Society of Anesthesiologists-physical status
%VC: (measured vital capacity/ predictive vital capacity) × 100 (%)
%FEV1.0: (forced expiratory volume in one second/forced vital capacity) × 100 (%)
NLR: Neutrophil-to-lymphocyte ratio
PLR: Platelet-to-lymphocyte ratio
CAR: C-reactive protein-to-albumin ratio
PNI: Prognostic nutritional index (10× serum albumin (g/dL) + 0.005× lymphocytes (/µL))
DSS: Disease-specific survival
OS: Overall survival
O group: Open surgery
L group: Laparoscopic surgery
